# The Royal Portsmouth Hospital

**Published:** 1912-03-30

**Authors:** 


### The Royal Portsmouth Hospital.

We are indebted to the *Portsmouth Times* for the substance of the following report of the proceedings at the meeting of the governing body of the Royal Portsmouth Hospital, held on the 22nd instant, Mr. Harold Pink in the chair. It was decided to take action upon the resolution of the Governors empowering the governing body to suspend the operation of rules 76 and 77 for such period as they considered desirable. Mr. Rundle again showed ability in dealing with this question, which resulted in the adoption of a suggestion by Mr. Cook to suspend the rules in favour of Dr. MacEldowney until such time as in the opinion of the Governors the special circumstances referred to in the resolution passed at the last subscribers' meeting should cease to exist. The Chairman is reported to have made a speech in reply to the criticisms of the institution in a leader in The Hospital of the 9th instant. Mr. Pink said that those criticisms might tend to do their hospital harm, and he was sure that if the Editor was acquainted with the way in which their institution had been worked for the past fifteen or twenty years he would have recognised that his strictures were quite unwarranted. He pointed out that Portsmouth differed very greatly from ordinary towns, especially the great manufacturing towns of the North; and their hospital, therefore, was carried on in different circumstances from those which usually obtained. They might regard the Dockyard as the equivalent of the factories, but the owners of the latter gave large subscriptions, whereas they only received a small sum from the Government. Their population, too, was not of a permanent character, and yet, comparatively speaking, the amount subscribed annually was above that which was obtained in the manufacturing centres, though their income was not so large, and they had few really rich people in their midst. In 1891 their funded property amounted to £19,673, in 1901 it was £24,518, and last year it was £25,864, notwithstanding the fact that stock to the amount of £5,176 had been sold out for various purposes. The hospital was really in a much better position to-day than it was ever in before, and he regretted the tone of the article, because it might have a detrimental effect on the status of their nurses. The Editor of The Hospital visited the institution in 1909, and then wrote congratulating the Secretary upon the good work which was being done, and the general appearance of the place. If he would come down again he would, he (the speaker) was sure, be convinced that they were doing the very best they could under the circumstances. When the King Edward Memorial Fund was completed they would be able to make an effort to increase their annual subscriptions. Meanwhile, he wished that the ministers of religion would endeavour to raise the Sunday Fund's contribution up to £1,000.

[We shall take the earliest opportunity to accept the invitation of Mr. Pink, the Chairman of the Governing Body, to inspect once more the Royal Portsmouth Hospital.—Ed., The Hospital.]

